# Chemical Composition, Nutrient Quality and Acceptability of Edible Insects Are Affected by Species, Developmental Stage, Gender, Diet, and Processing Method

**DOI:** 10.3390/foods10051036

**Published:** 2021-05-10

**Authors:** Victor Benno Meyer-Rochow, Ruparao T. Gahukar, Sampat Ghosh, Chuleui Jung

**Affiliations:** 1 Department of Genetics and Ecology, Oulu University, SF-90140 Oulu, Finland; 2 Agriculture Science and Technology Research Institute, Andong National University, Andong 36729, Gyeongsangbuk, Korea; sampatghosh.bee@gmail.com; 3 103, Niyogi Bhavan, Abhyankar Marg, Dhantoli, Nagpur 4400012, Maharashtra, India; rtgahukar@gmail.com; 4 Department of Plant Medicals, Andong National University, Andong 36729, Gyeongsangbuk, Korea

**Keywords:** entomophagy, insect edibility, insect farming, insect diversity, acceptability, nutrients, food security, diet

## Abstract

Edible insects have been considered as either nutritious food itemsper se, or as wholesome ingredients to various dishes and components of traditional subsistence. Protein, fat, mineral and vitamin contents in insects generally satisfy the requirements of healthy food, although there is considerable variation associated with insect species, collection site, processing method, insect life stage, rearing technology and insect feed. A comparison of available data(based on dry weight) showed that processing can improve the nutrient content, taste, flavour, appearance and palatability of insects, but that there are additional factors, which can impact the content and composition of insect species that have been recommended for consumption by humans. This review focuses on factors that have received little attention in connection with the task to improve acceptability or choice of edible insects and suggests ways to guarantee food security in countries where deficiencies in protein and minerals are an acute and perpetual problem. This review is meant to assist the food industry to select the most suitable species as well as processing methods for insect-based food products.

## 1. Introduction

Entomophagy (the habit of eating insects) has been practiced since time immemorial by humans [1,2,3] and their primate relatives [4,5]. Although entomophagy was not new to science, it was a paper by Meyer-Rochow [6], which for the first time suggested that edible insect species ought not to be neglected in the quest to safeguard future global food security. At present edible insects are still recognized as a sustainable food item by many residents of sub-Saharan Africa, South and Central America (including Mexico), South-East Asia and the Australia Papua New Guinea region. The consumption of insect species depends upon availability/access, suitability, preference, nutritional value, religious beliefs and social customs [7,8,9,10,11,12].

In North-East India, some highly appreciated species of edible insects are available (mostly seasonally) for sale at the local markets, but their cost is often higher than that of conventional animal meats or food of vertebrate origin [13,14]. This holds true also for Laos [15], Cameroon and many other African countries [16,17]. Nonetheless, the local people prefer the insects because of their taste and for traditional aspects [13,14,18]. However, insect consumption is declining, with one of the reasons being a shortage of the product due to a lack of facilities to efficiently and systematically rear suitable species and another reason in developing countries being an increasing “westernization” in terms of food choices [19]. As a result, sellers experience disruptions and delays in obtaining supplies and potential buyers are frustrated by the fluctuations of the product’s condition and availability.

Insects contain easily digestible quality protein with all the essential amino acids readily identifiable (except for methionine and tryptophan, which are present in low levels). The absence of tryptophan and fractional recovery of methionine and cysteine are attributed to methods of analysis and not necessarily because they are actually absent. For example, based on the data of 5 insects, viz. yellow mealworm *Tenebrio molitor* L., house cricket *Acheta domesticus* L., superworm *Zophobas morio* Fabricius, lesser mealworm *Alphitobius diaperinus* Panzer and the roach *Blaptica dubia* Serville, Yi et al. [20] observed that the amount of essential amino acids (EAA) was high and that the content of protein was similar to that of conventional meat products. In China, the pupal powder of the silkworm *Antheraea pernyi* Guerin-Meneville is appreciated, because of its substantial amount of protein (71.9%), EAA, fat (20.0%) and ash (4.0%) [21]. Information on the composition and content of nutrients in edible insects is readily accessible through journals, special reports and dissertations and has been summarized repeatedly [22,23,24,25,26].

With established techniques such as HPLC (high-performance liquid chromatography) for extraction and quantification of nutrients and bioprospecting of new species of edible insects, studies on the nutritional value of insects are being intensified with an aim to search economic and efficient ways to supply processed insects [27]. Currently, nutritional contents are not yet known for the majority of the surveyed/collected insect species of the various geographical locations and eco-zones that they occur in. Furthermore, knowledge and perception of factors that are encountered during the rearing of domesticated insects or those collected in the wild is limited and available only for a certain number of species. More studies on the chemical compositions of edible insects in relation to factors like geography, climate, processing and preparation methods would facilitate the identification of species most suitable for mass rearing and a potential to ameliorate the state of health in humans in certain parts of the globe [28]. This is especially important in view of the fact that most insects used as food today may not be much better nutritionally than traditional meats and that their ‘value’ is actually more related to environmental issues rather than their nutritional content [29,30].

This review examines and summarises a variety of factors that either are known (or have the potential) to influence an insect’s chemical composition and its nutritive value, such as a species’ taxonomic position or ecotype, thereby enhancing or reducing its acceptability as a food item for humans. The review illuminates in particular the roles that developmental stages, castes, an insect’s habitat and diet (whether natural or laboratory based) play in relation toaninsect’s amino acid and fatty acid content profile and to what extent the amounts of fibre, soluble carbohydrates and minerals depend on environmental factors. We highlight the importance of different processing methods, the risks of contamination and allergies and relate such factors to consumer choice as well as general acceptability of edible insects and insect-containing products as an alternative to conventional food items.

## 2. Nutrient Contents

### 2.1. Biological Factors: Insect Species, Developmental Stage, Sex and Caste, Organ and Ecotype or Biological Variants

#### 2.1.1. Insect Species

Edible insects generally belong to eight orders namely Blattodea (cockroaches, termites), Coleoptera (beetles), Diptera (flies), Hemiptera (true bugs), Hymenoptera (ants, bees and wasps), Lepidoptera (butterflies and moths), Odonata (dragonflies, damselflies) and Orthoptera (grasshoppers, crickets and locusts). Table 1 represents proximate nutrient composition of selected representative edible insect species and is not a compilation of all edible insect chemical analyses published to date. The results are based on dried insect samples, with the exception of the beetles *Oryctes boas* and *Oryctes rhinoceros*, which were not fully dry when analysed. The wide variation in the nutrient content among insect species generally depends on a variety of factors, of which geographic and climatic conditions as well as the insects’ food intake seem to be the most important factors. Although not to a very great extent, chemical content can indeed vary among different species belonging to the same genus (Table 2). The feeding regime, physiology and even ecological factors are more important determinants of the nutrient content than the species’ taxonomic proximity.

Table 3 contains comparative data on the amino acid composition of edible insect species. The results reveal that species belonging to the same genus may possess only somewhat different amino acid contents. To cite an example: various species of *Vespa* were found to differ with regard to the quantities of their amino acids [31,32]. Similar observations were made in connection with *Apis* spp. [33,34,35]. Moreover, the differences were attributed to the rearing system, including the insects’ feed and ecological condition. Palm weevils were found to have slightly different protein and amino acid content, depending on where they had been collected from [22]. However, irrespective of the amounts, the relative distribution of the amino acids was found to follow an almost identical trend in all of the individuals.

Overall, glutamic acid was always found to be most abundant, but among the essential amino acids leucine predominated followed by lysine. Although the scope to discuss the nutritional benefits of individual nutrient is limited in the present manuscript, it is worth mentioning that lysine content of edible insects is advantageous as it is often limiting in the cereal-based diet of humans. Species-specific fat and fatty acid content was apparent in edible insects (Table 4). In general, palmitic acid followed by stearic acid was the predominating fatty acids among the saturated kinds (SFA), while oleic acid was the most abundant among the monounsaturated fatty acids (MUFA). Species-specific patterns were noticed for mineral content (Table 5). However, the differences in the mineral contents are primarily attributable to geographic and ecological factors as the minerals are not synthesized in the animal body but are obtained from the dietary sources.

#### 2.1.2. Developmental Stage

Table 6 contains comparative data on the proximate nutrient content of different developmental stages of selected edible insect species. As already briefly touched upon, the contents of an insect can vary between adults, larvae, pupae and nymphs with regard to carbohydrates, protein, fat, fiber, ash, and minerals and there are complex reasons for this. In general, the protein content was found to be higher along with the more mature developmental stages. The opposite held true for fat content. Larvae and adults may feed on different foods and pupae usually do not consume any food at all, which would explain the differences in amino and fatty acid content as well as minerals seen in the different developmental stages of, for example, the honey bee [33]. To cite an example, in male bees (known as drones), the compositions of amino acids, protein and minerals all increase with development stage [61]. Saturated fatty acids dominated over monounsaturated fatty acids in the pupae but the reverse was reported for adults [61]. Variations in the nutrient composition of different post-embryonic developmental stages of nymphs and adults of the grasshopper *Zonoceros variegatus* were studied by Ademolu et al. [62]. Increments in protein but reductions in the amounts of fat from nymphs to adults were observed. Similar results, i.e., higher protein and lower fat content in parallel with the developmental stages hold true for three Blattodea species, namely *Blaptica dubia*, *Blaberus discoidalis* and *Blatta lateralis* [63].

The need to build up muscle tissue can change during developmental stages and is influenced by events of an insect’s life cycle. For example, termite worker adults may have less fat than the more sedentary nymphs and that is presumably because of their more active lifestyles and the greater need to burn fat to meet the energy demands of their leg muscles. The sedentary termite queen, as the only egg producer of the colony, would require much less muscle tissue, but considerably more fat. When reared for 13 weeks, cricket (*A. domesticus*) nymphs contained 36–60% crude protein and 12–25% fat with maximum amounts of palmitic, oleic, linoleic, linolenic and a small amount of arachidic, EPA (eicosapentaenoic acid) and DHA (docosahexaenoic acid) fatty acids [64]. The concentrations of Mg, Ca and Zn reached their optimum after 9 weeks when they were 1.30–11.30 mg, 1.40–19.70 mg and 0.20–16.60 mg/100 mg, respectively. On that basis, Kipkoech et al. [64] suggested cricket harvesting to occur preferentially between 9–11 weeks, because only at that age the larvae are in their nutritionally best condition for consumption [65].

In this context another issue is related to differences between developmental stages, specifically in relation to their life cycle physiologies: during events like droughts and overwintering periods the conditions of an insect may change. Ghosh et al. [52], for example, demonstrated changes in the bodycomposition of bumblebee (*Bombus terrestris*) queens during overwintering and summer periods.

#### 2.1.3. Sex and Caste

The powder of male silkworm (*B. mori*) pupae contained less protein than that of female pupae (reviewed by Mahesh et al. [72]), but there was no difference in the kinds of amino acid present between the two sexes [73,74]. Research by Cai et al. [75] and Kiuchi et al. [76] on male and female silkworm pupae has confirmed the presence of sex-related differences, adding information to the earlier reported differences in the amounts and compositions of the lipids in male and female pupae [77]. For example, more fatty acids were present in male than female pupae, but total lipid content of *B. mori* male pupae, on a fresh weight basis, was less (4.8%) than that of female pupae (9.0%) [78]. The content of unsaturated fatty acids was nearly the same in both sexes, but unsaturated acids were proportionately higher in female pupae [77]. In the case of *A. domesticus*, females have been shown to possess more lipids on a dry weight basis (18.3–21.7 g/100 g) than males (12.9–16.1 g/100 g), but less protein (63.1–65.7 g/100 g versus 69.9–71.9 g/100 g for female and male respectively). There was, however, no difference between the sexes with regard to the presence of essential amino acids (EAA) (72.3–77.1%), thrombogenicity (1.22–1.45%) and atherogenicity indices (0.53–0.58) [79].

In the subterranean termite, *Reticulitermes* sp., the reproductive caste had higher contents of the following nutrients than workers e.g., carbohydrates 2.7% versus 1.3%, protein 87.3% versus 81.7%, and amino acids 6.7% versus 4.7% [80]. Ntukuyoh et al. [81] evaluated the nutrient contents of the soldiers, workers and queens of the termite *Macrotermes bellicosus* in the Niger Delta region of Nigeria. Considering the average of the values provided, soldiers had the highest amount of protein (55.6%), lipid (2.7%) and fibre. Workers were especially rich in carbohydrates (65.1%), vitamin C (1.1 mg/kg), Fe (54.3 mg/kg), Mn (22.4 mg/kg) and Ca (58.3 mg/kg) whereas the queen contained higher amounts of vitamin A (7.0 mg/kg), Na (69.1 mg/kg), Mg (47.8 mg/kg) and Zn (25.2 mg/kg). Workers and queen had nearly the same amount of Cu (18.8 and 18.3 mg/kg respectively). In general agreement with this study by Ntukuyoh et al. [81], the same species collected from southwestern Nigeria by Idowu et al. [82] differed somewhat because of its higher content of ash, crude fibre, crude protein and carbohydrates in soldiers and workers rather than the reproductive caste which, on the contrary, had a higher fat content.

The weaver ant (*Oecophylla smaragdina*) exhibited a higher content of total lipid (average of annual values for larvae: 168.5, pupae: 140.7, and adults: 140.6 mg/g) in the queen while worker castes contained slightly more than half the amount (larvae: 112.0, pupae: 111.6, and adults: 100.8 mg/g) [83]. Lower contents of protein (37.5%) and ash (3.0%), but higher lipid content (36.9%) in the queen than that reported for other castes of weaver ants (presumably worker caste), were reported [84] for weaver ants in Thailand. In honey bees, considerable differences with regard to amino and fatty acids were documented not only for different developmental stages like larvae, pupae and adults, but also for the different sexes of the bees [33,61].

#### 2.1.4. Organs

Dué et al. [85] analyzed oil extracted from the integument and the digestive tract fat content (DFC) of the larvae of the South American palm weevil *Rhynchophorus palmarum* (L.). Fat content obtained from the integument (=“skin” in that paper) was lower (35.2%) than the DFC oil (49.1%). Oleic acid was highest in both oils (45.6–46.7%) followed by palmitic acid (39.9–40.4%). Saturated fatty acids were 45.1% and 45.0% for skin oil and DFC oil, respectively. Vitamin-A was found only in DFC oil. Regarding quality properties, the oil obtained from the integument was considered superior to that of the DFC oil, judged by their respective indices of iodine of 51.2 versus 48.4, peroxide of 6.9 versus 0.0 and oleic acidity (7.8 versus 0.6). However, data on the nutrient compositionsbased on specific insect organs such as fat body, ovaries, compound eyes, glands, etc.are extremely limited.

#### 2.1.5. Ecotype or Biological Variations

When *Rhynchophorus phoenicis* larvae were obtained from plantations of the raffia palm (*Raphia* sp.), yellow larvae contained more fat (27.7%) than white wild (22.2%) or white breeding (17.4%) larvae, more protein (8.8% for yellow wild versus 7.8 and 8.7% for white wild and breeding kinds, respectively), more carotenoids (805.0 versus 391.0 and 276.0 µg/100 g for yellow wild, white wild and breeding respectively), but less polyunsaturated fatty acids (PUFA) (0.5 versus 0.8%) and tocopherol (2.3 versus 4.8 and 4.1 mg/100 g for yellow wild, white wild and breeding kinds, respectively) [86]. In Uganda, no significant differences were recorded for the dry matter and moisture contents between the brown ecotype and the green one of the cone-headed grasshopper, *Ruspolia nitidula* (Scopoli). High potassium content (5.55 mg/kg) was recorded [87] in brown grasshoppers collected in Kampala during the March–May season but not in the November–December season. These findings provide some information on the likely correlation between colour change (as a result of the ecotype) and chemical composition in grasshoppers influenced by climatic conditions and geographic location.

### 2.2. Ecosystem and Insect Habitat

Sustainability in insect diversity and year-round (or at least seasonal) availability is important for family livelihood of local communities [17]. Terrestrial insects are more abundant and easier to collect than aquatic species and may therefore be recommended for consumption in preference to the latter [88]. On the other hand, Williams and Williams [89] demonstrated the potential of aquatic insects to contribute to human diet. In a study of nutrient contents in both aquatic and terrestrial insects by using linear models, Fontaneto et al. [88] showed that terrestrial insects contained a significantly lower amount of monounsaturated fatty acids (22.5%) than that reported for aquatic edible insects (33.8%). In contrast, a higher amount (44.2%) of PUFA was found to be generally present in terrestrial insects rather than the aquatic species (27.9), although statistically the difference did not reach significance.

In Uganda, the effects of two swarming seasons (March–May and November–December) on the nutrient contents of *R. nitidula* were studied. No significant differences were found in the comparative contents of protein (39.7–40.4% in the March–May season and 37.0–39.1% in the November–December season), fat (41.9–42.4% in the March–May season and 41.2–43.0% in the November–December season) and carbohydrates, but carotenoids (2084.8–2273.1 µg/100 g in the March–May season versus 913.7–1389.4 µg/100 g in the November-December season) and fibre (11.3–12.2% in the March–May season versus 13.1–14.3% in the November–December season) differed significantly with the seasons [87]. In an additional study, Ssepuuya et al. [90] reported that the geographical area was highly influential with regard to the insect’s mineral content within a season, whereas the season alone affected significantly the variation in the contents of protein (34.2–45.8%), fat (42.2–54.3%), ash and minerals in *R. nitidula*. Geographical area or season, however, were not seen to affect the compositions of amino and fatty acids.

Maximum contents of fat in *O. smaragdina* ant queen larvae (249.2 mg/g), queen pupae (228.9 mg/g), worker larvae (129.9 mg/g), worker pupae (133.1 mg/g) and worker adults (123.2 mg/g) from Assam (India) were recorded in March, whereas maximum fat (207.3 mg/g) in queen adults was found in April and minimal amounts occurred during the November–February months [83]. These data, which were valid for various sites, can be used by collectors to decide the most suitable period to collect preferred insect species and their life stages based on nutrient contents.

In Botswana, Madibela et al. [91] studied contents of *Imbrasia belina* larvae sampled at three eco-sites (Mauntlala, Moreomabele, Sefophe). At Moreomabele, a high content of acid detergent fibre (ADF) (230.9 g/kg dry matter) and acid detergent insoluble nitrogen (ADIN) (18.0 g/kg DM) was noted, whereas these contents were least (155.5 g/kg for ADF and 11.8 g/kg for ADIN) at Sefophe. At Mauntlala, contents of ADF and ADIN were 175.1 and 12.2 g/kg respectively. In *M. bellicosus* termites from Nigeria, Idowu et al. [82] reported highest contents of vitamins (A, the B-complex, and C) in reproductive castes collected from farmland, whereas those from an industrial estate had the highest amount of Cu (0.076 mg/L). Lead was detected only in the soldiers. The highest value of Cr in workers was found in termites collected from farmland (0.226 mg/L) and a waste dumping site (0.223 mg/L). The chemical composition between hibernating queen bumblebees, *Bombus terrestris* (L.), during the winter season differed significantly from summer queens and featured an increased ratio from 3.6 to 4.9 between unsaturated and saturated fatty acids [52]. Thus, differences may be observed in species even if collected from a single site, but at different times of the year.

### 2.3. Insect Feed

Insects are reared on a synthetic diet (containing only chemical ingredients), semi-synthetic or meridic diet (containing synthetic and plant material). Both categories of food are classified as laboratory diets. The natural diet comes solely from natural sources, i.e., host plants or plant products. More details about these diets can be found in [92].

#### 2.3.1. Plant Material

The nutritional status of the host plants that insects feed on affects the nutrient contents of the edible insects. When *vermiwash* (water washings of earthworm cocoons) at 10%, 25% or 50% was sprayed on mulberry leaves and fed to fifth instar *B. mori* larvae, significant increases on a dose-dependent basis were observed with regard to carbohydrates, protein and fat [93]. However, when Ebenebe et al. [94] reared *R. phoenicis* larvae on four organic substrates, e.g., sugarcane tops, split watermelon, split pineapple and raw papaya, their larvae had (on dry weight basis) normal carbohydrate, protein and fibre, contents that did not vary much and statistical significance was lacking. They concluded that split pineapple can be selected for feeding larvae as a potential source of protein and fluid.

Among 10 African host grasses of the longhorn grasshopper *Ruspolia differens* Serville, Malinga et al. [95] noted maximum survival of 65% on *Chloris guyana*, *Pennisetum purpureum*, *Setaaia sphacelata*, *Brachiaria ruziziensis* and *Sporoborus pyramicloris*. Fresh weight was highest (0.383 g/adult) on *P. purpurium* and *B. ruziziensis*. On a mixed diet basis, significantly shorter development time (16 days) from nymph to adult and higher survival (>65%) occurred in diversified diets compared to the use of single grass species. Contents of PUFA and fatty acid composition did not differ significantly among the diets. Therefore, a mixed feed can be recommended for mass rearing of grasshoppers.

Quaye et al. [96] evaluated four diets based on the oil palm yolk (*Elaeis guineensis* Jacq.) alone or mixed with banana and pineapple waste or millet waste. The highest protein (32.0%) and fibre (8.4%) content was found in *R. phoenicis* larvae fed on oil palm yolk. Practically, year-round non-availabilities of some plant materials and the costs involved to procure them make vegetative substrates often uneconomic for mass production by farmers and tribal communities [94].

Whenever an insect’s natural food is altered, nutrient contents may be affected. For example, adding wheat bran to grass (natural food) to feed the locusts (*Locusta migratoria*) influence the nutrient composition, the protein content in adults varies from 555 g to 649 g/kg (dry matter) and the fat content varies from 186 g to 296 g/kg [97]. While rearing the Asian palm weevil, *Rhynchophorus ferrugineus* (Olivier) on three substrates, Cito et al. [98] recorded total fat of the order of 57.6%, 58.4% and 60.0% for apple juice, pineapple palm (*Phoenix canariensis* Chaubaud) and cocoa palm [*Syagrus romanzoffiana* (Cham.) Glassman] respectively. For the three substrates, the content of monounsaturated fatty acids was 43.9%, 41.6% and 44.7% while the unsaturated fatty acids reached 56.1%, 57.2% and 52.8% of the total fatty acids, respectively [98].

Larvae of *R. ferrugineus* fed on raffia palm [*Raphia farinifera* (Gaertn.)] were heavier (159 g) than those reared on oil palm, which weighed only 52 g [99]. These findings demonstrated how different substrates can be used and can be practical in relation to weevil rearing. Feeding neonate nymphs of the grasshopper (*R. differens*) till the imago stage on inflorescences of local grasses (8 species) did not strongly modify fatty acid content or composition or even adult body weight [100]. The ratio of n-6:n-3 fatty acids was generally low, a point of note in connection with the need for a healthy human diet. Significant differences in the composition of rare fatty acids (n-6/n-3, arachidonic acid, alpha-linolenic acid), however, were present in the grass species. In another study, Rutaro et al. [101] used inflorescences of four plants and found a high content (21%) of essential fatty acids in field-collected sixth instar nymphs compared to the low content (12–13%) in connection with less diversified diets. However, total lipid content and weight of grasshoppers did not differ among diets, but it showed that the fatty acid composition can be influenced by an insect’s food uptake.

#### 2.3.2. Laboratory Diets

Laboratory or artificial diets have certain advantages over natural plant material for rearing silkworms, because such diets are semi-synthetic or synthetic and can be used for several insect species. They help to rear insects that vary little between individuals which can then be made available whenever needed for bioassays or other purposes [92,102].

Rutaro et al. [103] formulated artificial diet for *R. differens* containing rice seed head, finger millet seed head, wheat bran, chicken egg buster, sorghum seed head, germinated finger millet, simsim cake, crushed dog biscuit pellet and shea butter. More diverse diets resulted in increased content of PUFA and linoleic acid. Fatty acid composition differed significantly among the diets. The workers concluded that essential fatty acid content can be increased by feeding grasshoppers on highly diversified diets, particularly when mass rearing is the objective.

Ghaly [104] prepared a diet of dry ingredients (corn flour + whole wheat flour + wheat bran + dried yeast powder mixed in a ratio of 3:3:3:1, by weight) and liquid ingredient (glycerine + honey mixed in 1:1 ratio, by weight). The two ingredients were then mixed in a 1:1 ratio. This diet proved superior to plant material in rearing *Gonimbrasia* *belina* and *Anthoaera zambezina* in Zambia. In Nigeria, the *G. belina* larvae reared on a semi-synthetic diet (corn starch + vegetable oil + glucose + cellulose + mineral mix + vitamin mix + protein) contained 7.1% carbohydrates, 35.2% protein, 15.2% fat and 7.4% ash [105]. These contents were comparatively low in larvae fed only natural plant biomass [105]. These diets should be tried in connection with other lepidopteran larvae consumed in Africa. Stull et al. [106] successfully reared *T. molitor* on a diet containing 40% stover (corn by product) by weight. Analyses after 32 days into rearing showed that the larvae contained all the essential amino acids. In another experimental series, the insects completed metamorphosis and all larvae survived on a 100% stover diet for multiple generations. Therefore, this diet can be recommended for the rearing of *Tenebrio* and possibly some other beetle species in the laboratory.

Indoor rearing can be further improved by fortifying the laboratory diet. For example, De Wit [107] prepared a vitamin D-enriched diet to rear *B. mori* larvae. There were significant changes in the content of the macro nutrients compared with diets that had not been fortified, e.g., increases in protein (61.2% versus 58.8%) and reduction in fat (37.3% versus 39.5%). The addition of the commercial protein supplement Nutrilite^®^ increased the content of sericin and fibrous protein by 68% and 56%, respectively, with addition of 10% supplement compared to no addition for the late larval instars of *B. mori* [108]. This finding implies that laboratory diets should be preferred for edible insects if the objective is to obtain a greater amount of nutrients from the insect biomass.

#### 2.3.3. Plant Based by-Products

While assessing 18 diets based on industry by-products (such as, potato protein, barley mash, leftover of turnip-rape and broad beans) for rearing of crickets such as *A. domesticus* and *G. bimaculatus*, Sorjonen et al. [109] reported yields of *A. domesticus* as high as 4.10 g on barley mash and 5.12 g of *G. bimaculatus* on turnip rape. The average weights of female and male *A. domesticus* were 0.459 g and 0.342 g, respectively, whereas in the case of *G. bimaculatus* the corresponding weights were 0.912 and 0.626 g. Thus, these protein-rich products can replace currently-used soybean in mass rearing. Further, Sorjonen et al. [110] added two more diets (mix of broad bean and pea, mix of potato, carrot and apple) for rearing of *R. differens*. Increasing protein level in the diet up to 17% enhanced growth, development time, and survival. Fatty acid content and composition differed as per diet. For example, high PUFA content was noted in connection with barley mash, barley feed and broad bean diets. Fresh weight was highest (0.507 g/adult) in the Suomalainen diet with vitamins and minerals as supplements [110]. However, the study suggested that the best options were barley feed, barley mash or potato protein.

Lehtovaara et al. [111] recorded nearly 10-fold increased contents of linoleic, alpha-linolenic, eicosapentaenoic and docosahexaenoicfatty acids in *R. differens* adults when these acids were present in the artificial diet. Development performance was also improved with n-6/n-3 acid ratio. Lack of protein and fat in the diet prolonged development and resulted in low final weight. Therefore, it is necessary to design nutritional content in artificial diets to obtain heavy, nutritious grasshoppers through mass rearing.

### 2.4. Insect Processing and Product Quality

Traditionally, insects are consumed raw or processed (dried, crushed, pulverized, ground, pickled, cooked, boiled, fried, roasted/grilled, toasted, smoked or extruded [112]. Besides these techniques, Kewuyemi et al. [113] suggested fermentation to enrich the inherent composition of insect-based products and to induce anti-microbial, nutritional and therapeutic properties. Similarly, defatted *T. molitor* larvae and oil could be used as food ingredients [114]. Defatted mealworm powder contains sufficient protein, minor amounts of minerals and bioactive compounds and has a savory taste due to plentiful amino acids. Oil is abundant in γ-tocopherol and possesses good shelf life [114].

Before processing, insects are often kept without food for fasting and large specimens are degutted or defatted, because the gut may contain undigested plant material, excreta, microbes etc.; moreover, degutted insects have higher contents of crude fibre protein [91,112]. This practice, being efficient and practical, has been routinely adopted by tribal communities particularly for large lepidopteran larvae. Processed insects can be preserved by freeze-drying or sun-drying and in canned form. Processing methods may differ as per consumer preference, availability and suitability of insect species, social custom, religious rituals, tribal ethics and family tradition [17]. The effects of four different drying temperatures (80, 100, 120, 140 °C) on antioxidant properties of silkworm powder was studied by Anuduang et al. [115] and showed that the lowest drying temperature preserved phenolic compounds and antioxidants best.

In selecting a food item on the basis of “post-ingestive fitness”, the processing method can help to remove anti-nutrients and other unhealthy components as well as increasing the shelf life. Thus, processing is important to maintain the level of nutrient content, to extend the shelf life and to obtain functional and fortified foods [112]. In food processing units, products are enriched with insect chitosan (a polysaccharide derivative of chitin), which is more soluble and therefore preferred over raw chitin in food processing [116]. Local communities frequently know methods to improve insect-based foods with traditional wisdom built on generations of experience [117].

Methods can of course change and be replaced by others, because each method has certain advantages or disadvantages suiting regional needs.For example, roasting, cooking and frying are largely employed in North-East India, because of the better taste of insects compared to boiling and baking [13]. Generally, vitamins are susceptible to heating and heat processing decreases the level of these vital compounds entirely or partially. Storage conditions are important to prevent the deterioration of insects. However, tocopherol content in *T. molitor* and *Zophobas mario* L. was not altered under different environments [118], but antioxidant properties in silkworm powder were [115]. Nyangena et al. [119] examined the effects of traditional processing techniques, i.e., boiling, toasting, solar-drying, oven-drying, boiling + oven drying, boiling + solar-drying, toasting + oven-drying, toasting + solar-drying on the proximate composition and microbiological quality of *Acheta domesticus*, *Ruspolia differens*, *Hermetia illucens* and *Spodoptera litoralis*. They found that traditional processing improved microbial safety but altered the nutritional value. Moreover, species- and treatment patterns clearly existed. A few examples below may demonstrate the different processing techniques used in selected insect species.

#### 2.4.1. Lepidoptera

Maceration of *Bombyx mori* pupae, being simple, practical and not at all costly, is the most common method employed by indigenous people. For example, Winitchai et al. [120] reported 72–79% of unsaturated fatty acids and 32–44% of alpha-linolenic acid in Soxhlet extractions of fresh pupae whereas respective contents after maceration were 75–80% and 40–46%. Defatting of pupae and turning them into a powder is practiced to retain nutrients as witnessed in comparisons with the powder of non-defatted pupae [121], e.g., crude protein 57.4% versus 48.3%, digestible protein 48.3% versus 40.1%, soluble protein 29.0% versus 16.4% and carbohydrates 15.8% versus 10.2% [74].

In India, fresh pupae of a bivoltine breed of *B. mori* contained 17.1% protein and 9.2% fat compared with 56.9–75.2% protein and 24.9% fat of dried pupae [122]. Oil extracted from pupae is an important source of unsaturated fatty acids (75%), essential linoleic acid (33%) and alpha-linoleic acid (35%) [121].

Once the silk threads are separated, silkworm pupae (often referred to as chrysalis) become waste matter, but the powder of the empty pupae contains important nutrients [72] as also reported by Rao [123] from India, e.g., 48.7% protein, 30.1% fat, 8.6% ash, and significant amount of minerals and vitamins; however, defatted spent pupae contained higher protein, e.g., 75.2%. In Brazil, Pereira et al. [124] recorded that chrysalis (pupae) toast contained protein (51.1%), fat (34.4%), linolenic (24.4% of total fatty acids), palmitic (24.6% of total fatty acids), stearic (7.6% of total fatty acids), oleic (34.8% of total fatty acids), and linoleic acids (7.0% of total fatty acids), Zn (244.0 µg/g) and K (4.8 mg/g). Because of the high content of major nutrients and essential fatty acids, chrysalis toast was recommended as an alternative dietary supplement in Brazil [124]. These results suggest that as far as possible, larvae should be defatted and de-oiled before processing. The powder is less perishable and can be stored in cool and dry places in a house/hut or refrigerator for a long time and may be consumed whenever needed. Currently, this processing technique remains invalidated although valuable for nutritional security. Furthermore, steamed and freeze-dried mature silkworm powder exhibits different pharmacological effects [125] as calorie restriction mimetics [126], including enhanced mitochondrial functions in the brain [127].

In Africa, when *Gonimbrasia* (=*Imbrasia) belina* larvae were degutted, washed and dried, the shelf life could be extended up to one year without any deterioration in quality [128]. Lautenschläger et al. [129] compared three traditional techniques of preparation before consumption, e.g., evisceration, cooking and drying of silk moth, *Imbrasia epimethea,*(Drury) larvae. No significant differences for protein, fat, amino acids and fatty acid composition were observed among the three treatments. Gut removal reduced carbohydrates originating from the leaves of the host plant and showed negative effects on the nutritional value. No changes in the content of nutrients (except reduction in MUFA) were observed when the larvae were exposed to thermal processing. The study by Medigo et al. [130] showed that processing can also involve adding foods containing high amounts of sugar and saturated fats, although such additions diminished the overall nutrient content of the insect product [130].

In Botswana, Madibela et al. [91] compared nutrient contents in *Gonimbrasia* (=*Imbrasia) belina* larvae without any processing and those that were degutted salted or degutted + salted. Degutted samples had a higher content of crude protein (567.5–579.3 g/kg DM) than non-degutted larvae (505.3–537.5 g/kg DM) but had a lower concentration of ash (41.2–39.0 g/kg in degutted versus 58.0–59.2 g/kg in non-degutted larvae). A similar trend was present with regard to acid detergent fibre (ADF) (148.5–173.2 g/kg in non-degutted versus 158.8–268.1g/kg DM in degutted larvae). The addition of salt increased ADF, whereas the degutted + salted samples had a higher ADF than degutted or salted larvae or those without processing. Unprocessed insects diluted the concentration of protein but increased the fiber and tannin contents.

It is possible that the heating results in the formation of new components, and that solvents used for the extraction of protein at industrial level can affect the safety of novel protein products [131]. In two edible insects namely *Imbrasia truncate* Aurivillius and *I. epimethea*, appreciated by consumers in the Congo river basin, Fogang Mba et al. [132] reported respective contents (fresh weight) of protein as high as 19.1 and 20.1 g and of fat reaching 6.8 and 6.7 g/100 g in the larvae. Unsaturated fatty acids in *I. truncata* and *I.epimethea* were 2.6 and 2.2 g/100 g, of which alpha-linolenic acid amounted to 1.9 and 2.2 g/100 g respectively. Processing procedures are held to be responsible for the ranges and, therefore, need to be well planned in connection with food products that contain insects.

#### 2.4.2. Coleoptera

There was a considerable increase in the content of carbohydrates, protein and fat of sun-dried larvae of *O. rhinoceros* and *R. phoenicis* over non-processed ones. Pith of coconut palm for *O. rhinoceros* [133] and raffia palm pith for *R. phoenicis* [134] served as natural feed source whereas a laboratory diet comprising of corn starch, vegetable oil, sucrose, glucose, cellulose, mineral mix and vitamin mix for *O. rhinoceros, Gonimbrasia (=Imbrasia) belina, M. bellicosus* and *R. phoenicis* [105] was successfully used. A greater increase in dry weight was seen in laboratory-diet fed larvae than those fed only natural plant material.

Wheat flour dough enriched with ground *T. molitor* larvae at 10% resulted in a softer dough with increased size and weight when baked at 200 °C for 22 min [135]. High moisture extruded meat substitutes can contain the biomass of another species of beetle, e.g., *Alphitobias diaperinus* (Panzer) 40% mixed with soy dry matter up to 60% [136]. Water content in the product was important for improving its physical properties, i.e., the sensation when biting or chewing it.

#### 2.4.3. Orthoptera

In laboratory experiments, *R. differens* adults were frozen at about −50 °C for 96 h [137]. Another lot was oven-dried at 60 °C for 24 h. No significant differences in the two methods were present for the content of average crude protein (46.4 for freeze dried and 47.7% for oven fried samples) and fat content (35.6% and 35.5% for freeze and oven dried samples, respectively). The content of chitin, by contrast, varied from 11.3 to 13.4% for freeze and oven dried samples. The mineral contents (in 100 g DM) in oven-fried and freeze-dried grasshoppers were as follows. Na = 54.0 versus 69.1 mg, K = 779.2 versus 816.4 mg, Ca = 895.7 versus 1034.7 mg, Mg = 145.8 versus 161.0 mg, Zn = 14.6 versus 14.2 mg, Fe = 216.6 versus 220.1 mg, P = 652.3 versus 685.9 mg, Cu = 1.7 versus 1.7 mg and Mn = 7.4 versus 8.3 mg [137]. On the contrary, toasting + drying significantly reduced protein digestibility in *R. differens* (76.4% versus 82.3% in fresh specimens of the grasshopper: [138]). Boiling of grasshoppers resulted in significant increases of protein and elements such as Fe, Zn, Cu, Mn and Ca contents, but decreases in the fat content on dry matter basis. Amino and fatty acid profiles were minimally affected but a significant reduction in ash content was noted. In case of roasting, there was an increase in Ca and trace mineral elements. The colour was uniformly intensified in green and brown polymorphs when roasted together. The aroma of heat-processed grasshoppers was influenced by lipid oxidation [139].

Fresh dried grasshoppers had a maximum fat content of 43.1% compared with only 16.3% in fresh insects. Also, there was a reduction in niacin content when the grasshoppers were toasted, toasted + dried or fresh dried (3.06–3.28 mg/100 g versus 3.61 mg in fresh insects [138]. High protein and fat contents, which contributed to >75% of the dry mass, justify the high reputation of this nutritionally valuable species for human consumption.

Hassan et al. [140] compared two processing methods for the tree locust, *Anacridium melanorhodon* (Walker) and reported that frying of adults resulted in slightly increased fat absorption (1.3 mL/100 g by frying versus 1.0 mL/100 g by boiling).Boiling, however, resulted in a reduction in tannin content (9 mg/100 g by frying versus 5.8 mg/100 g by boiling) and high protein digestibility (41.1% by frying versus 49.9% by boiling). Better digestibility was associated with water absorption (2.5 mL/100 g in fried versus 2.9mL/100 g in boiled insects). Farina [141] compared the broth prepared by cooking *A. domesticus* adults after freezing them with those adults that were alive when cooked. There was a significant difference in the pH, overall acceptance and perception of saltiness and umami/savory flavour. These qualities were associated with the breakdown of glycogen and the formation of lactic acid during the killing of the insect. Therefore, a proper processing method needs to be selected in connection with insect-based protein in the presence of sodium chloride.

#### 2.4.4. Blattodea

Kinyuru et al. [138] reported no significant change in protein digestibility (range of 90.1–90.5%) in the winged termite *Macrotermes subhyalinus* Rambur, but a significant increase in fat content was present due to fresh drying (42.3 g versus 19.8 g/100 g in fresh collection). A significant reduction in retinol content (0.98–1.6 µg/g versus 2.2 µg/g in fresh insect) and riboflavin content (2.8 mg/100 g in toasted termites versus 4.2 mg in fresh stock) was recorded [138].

When the flour of the soldier caste of the termite *Syntermes* sp. was mixed with honey spread at 8, 16 and 24% and then processed by pan-frying at 80, 90 and 100 °C, the 24% mixture exhibited a significant increase in the content of protein from 5.6 to 15.9 g/100 g, Fe from 3.8 to 8.8 mg/100g and Zn from 1.8 to 4.5 mg/100 g. It also led to improved sensory qualities, especially flavour and taste [142].

## 3. Insect Quality

### 3.1. Content of Anti-Nutrients

Table 7 represents anti-nutrient contents of selected edible insects based on currently available information. In comparison to the data on nutrient content of edible insects, data on anti-nutrients are limited and even controversial. By definition anti-nutrients hinder or inhibit the absorption of nutrients, especially minerals, but some may also provide anti-oxidants like polyphenols including tannins. Due to insufficient data, saponins, alkaloids, etc. have not been included in our list.

The available literature shows high variations in the amounts of individual anti-nutrient compounds. Edible insects are mostly herbivorous, feeding on plants and their parts. For self-preservation plants synthesize different types of secondary metabolites and these secondary metabolites are known as allelochemicals and accumulate in the bodies of plant matter-ingesting insects. Their primary action is to inhibit the absorption of necessary nutrients and they are therefore termed anti-nutrients. The wide variation of the insects’ anti-nutrient content is likely to be due to the different chemical compositions of plants on which the insects feed. Primarily, it depends on the environment and the site that a plant is growing. However, a systematic protocol has to be developed in order to quantify the anti-nutrient contents. In this context it is also worth mentioning that the development of rearing techniques of edible insects under controlled conditions can minimize or even avoid the contamination of insects with these allelochemicals.

### 3.2. Contamination with Chemical Pesticides, Inorganic Products and Infestations with Insect

Chemical pesticides sprayed on host plants of edible insects are often stored in the form of residue in the insect body. That chemical contamination causes a deterioration of, for example, the quality of edible insects such as the locust *Locusta* sp. in Kuwait (and the water bug, *Lethocerus indicus* in India was shown [149,150]. Poma et al. [151] reported low concentrations of heavy metals, DDT (Dichlorodiphenyltrichloroethane) and dioxins in edible insects compared with chicken egg, fish, and animal meat. In China, heavy metals have been found in *B. mori* larvae fed on mulberry leaves harvested from plants cultivated in soil treated with municipal solid waste compost [152] or grown in soil-polluted fields [153]. The hide beetle, *Dermestes maculatus* DeGeer, which feeds on dry animal matter, also attacks dry edible insects. In the laboratory, Fasunwon et al. [154] experimented with artificial inoculations by this beetle with larvae of the rhinoceros beetles *Oryctes boas* (Fabricius) and *R. phoenicis.* There were significant differences in the nutrient contents when containers were provided with a mixture of salt and pepper (10 g/container). Protected larvae of *O. boas* contained 56.1–60.6% protein versus 39.3% of the control and *R. phoenicis* had 34.8–37.3% protein versus 22.7% in the control. A similar trend was present for the fat content with 4.3–7.8% versus 4.5% in the control of *O. boas* larvae, and 20.1–30.7% versus 13.5% in the control of *R. phoenicis* larvae. Therefore, storage of well dried edible insects mixed with salt and pepper has been recommended to maintain the nutritional quality [154].

### 3.3. Microbial Contamination

Contamination with microorganism is a major factor in the deterioration of the quality of insect-based food items. Numerous bacterial species are known to affect insects including *Bacillus cereus* Frankland andFrankland, *Staphylococcus aureus* Rosenbach, *Escherichia coli, Rickettsiella* spp. Some insects also act as carriers of human pathogens of the genera *Salmonella, Campylobacter, Shigella* [155]. Additionally, grasshoppers serve as intermediate hosts to several avian parasites, horsehair worms and tapeworms [156].

In Ghana, larvae of *R. phoenicis* fed on raffia palm or oil palm contained bacteria at 1.3 × 10^7^–6.5 × 10^6^colony-forming units (CFU)/g body mass, which was higher than the acceptable level of 5.0 × 10^6^ CFU/g [99].In Nigeria, Braide andNwaoguike [157] assessed the quality of processed larvae of this widely consumed species and reported a load of bacterial and fungal counts of the order of 1.68 × 10^5^ CFU/g and 9.2 × 10^2^ CFU/g, respectively. The major bacteria were *Lactobacillus plantarum* Bergey, *S. aureus* Rosenbacch, *Bacillus subtilis* (Ehrenberg) Cohn, *Pseudomonas aeruginosa* (Schroter) Migula and *Proteus vulgaris* Hauser.

Major fungal species were *Cladosporium* sp., *Penicillium verrucosum* Dierckx, *Aspergillus flavus* Link and *Fusarium poae* (Peck) Wollenweber. In Zimbabwe, stink bugs such as *Encosternum deregorguei* Spinola, stored in dung-smeared wooden baskets, were found contaminated with aflatoxin (a carcinogenic mycotoxin: [158]. Consequences of the contamination were, however, not studied. Braide et al. [159] recorded contamination of bacteria (4.49 × 10^7^ CFU/g) and fungi (9.5 × 10^6^ CFU/g) from collections in the wild of caterpillars of the emperor moth, *Bunaea alcinoe* (Stoll) in South Africa; caterpillars harbouring bacteria such as, *P. aeruginosa* and *Proteus mirabilis* produced undesirable flavours in food products, and *S. aureus, B. cereus* and *E. coli* produced toxins [159]. Furthermore, *S. aureus* was easily introduced during the handling of insects [160].

Processing (by traditional and innovative methods) can eliminate microbes or at least considerably reduce their load [112]. In lactic acid fermentation of a mixture of sorghum flour and *T. molitor* larvae, the level of spore forming bacteria (*B. subtilis, B. megaterium, B. licheniformis*) remained stable suggesting the bacteria were unable to germinate; their quantity remained at the acceptable level of <10^3^ CFU/g [161]. Some of the effective and practical safety measures discussed below can be implemented. For example, thorough washing and heating can reduce microbial contamination to some extent [155]. Also, insect boiling before roasting proved effective to keep spore forming bacteria under check and insect dehydration can also reduce microbial contamination because at the lower humidity bacteria grow less.

Modified packaging systems are needed to prevent further contamination and to enhance shelf life of stored edible insects [159]. Regular monitoring and evaluation for bacterial contamination should be undertaken during storage as environmental changes can affect the insect quality. Therefore, refrigeration is employed to prevent contamination compared to outside storage at ambient temperature [162]. Packaging material is another critical aspect for safety. For example, boiled, solar dried and milled house crickets when stored for 2 months at ambient temperature in polypropylene (PP), plastic or polyethylene packages, the PP-packaged insects lasted only 45 days compared to 2 months with the other packaging materials. In all packages, iodine values, contents of SFA, MUFA and PUFA significantly decreased, peroxide, p-anisidine and saponification values increased and incidences of yeast and mould (*Aspergillus, Alternaria, Penicillium*) were high [162]. Although plastic packages with lids outperformed bags, adding a layer of polypropylene on the inner side can minimize permeability and exposure to both air and water vapour and thus can prolong the shelf life [162].

In food industries in Europe, a recent study has revealed antibiotic resistant genes in *A. domesticus.* As a preventive measure, Roncolini et al. [163] suggested standardization of the production processes and a prudent use of antimicrobials during the rearing of edible insects. Even though *Spiroplasma* sp. and *Erwinia* sp. in *T. molitor* and *Parabacteroides* sp. in the tropical house cricket, *Gryllodes sigillatus* (F. Walker) were the major pathogens during rearing of insects in the laboratory, Van der Weyer et al. [164] recommended that food safety should include also general bacteria like *Cronobacter* spp. or spoilage bacteria (*Pseudomonas* spp.) to be considered as potential human pathogens.

### 3.4. Allergenic Proteins

Allergenic reactions to the ingestion of insects and cross-reactivity with homologous proteins and co-sensitization between insects have been reported [165]. Allergens of edible insects identified as muscle proteins such as myosin, sarcoplasmic-Ca-binding protein, the major being tropomyosin and arginine-kinase (also known as pan-allergens).Persons who are allergic to dust mites and crustaceans could have an allergic reaction to foods containing *T. molitor* proteins [166]. It is possible that human susceptibility is due to immunoglobulin E-binding cross-reactions. For example, persons allergic to shrimps react with protein extract of *T. molitor* that shows Ig-E-binding cross-reacting allergens with other phylogenetically related groups of arthropods. Therefore, consumers allergic to shellfish should invariably be notified about the risk of developing an allergy by labelling the insect products accordingly [167].

It was shown that thermal processing and digestion did not eliminate insect protein allergenicity [168]. But the recent technique of high hygrostatic pressure coupled with enzymatic (pepsin) hydrolysis improvedin vitrodigestion of allergenic proteins of *T. molitor*. This technique can be an alternative strategy to conventional hydrolysis to generate a large quantity of peptide originating from allergenic *T. molitor* proteins [169].

### 3.5. Food Fortification

Insects as supplements for the predominant staples like corn, cassava, sorghum, pearl millet, beans and rice are commonly employed by indigenous folk in many developing countries. Insects also form a sustainable ingredient to produce new food items because fortification increases richness in nutrients. Corn being a major staple food crop in sub-Saharan Africa, members of local communities consume this tryptophan- and lysine-deficient product in considerable quantities. But this diet can be supplemented with termites and lysine-rich silkworms to overcome the deficiencies in these amino acids [170]. Similarly, natives of Papua New Guinea consume crop tubers with a low content of lysine and leucine. Vitamin deficiency can be remedied by supplementing the diet with *Rhynchophorus* spp. larvae, containing high amounts of vitamins and lysine. Tubers enriched with tryptophan and aromatic amino acids can render a diet more balanced and nutritious [170]. Ayensu et al. [171] mixed flour (70%) of *R. phoenicis* with orange-fleshed sweet potato and wheat flour to prepare biscuits. These biscuits had their energy, fat and protein content increased by fortification with palm weevil larvae powder compared with biscuits containing 100% wheat flour. Contents of Ca, Fe, Zn were also increased. The biscuits were highly appreciated by pregnant women in eastern Africa.

Winged termites such as *M. subhyalinus* added at 5.0% to cereal-based recipes in the Lake Victoria region of Kenya not only improved the food quality (protein, retinol, riboflavin, iron, zinc content) but made the food also more attractive [172]. In Mexico, corn bread (‘tortilla’) is sometimes supplemented with *T. molitor* larvae to improve consumer acceptance as well as nutritional content especially essential amino acids [173]. Likewise, Kwiri et al. [128] suggested that edible insect *G. belina* could be an alternative substitute of the current local plant-based supplements (beans, peanut, cowpea) in Zimbabwe.

Kim et al. [174] recommended up to 10% replacement of lean meat/fat portion with flour of the cricket *A. domesticus*. When compared with meat to which no cricket flour had been added, this level of mixing increased the content of protein from 14.0% to 20.7%, fat from 9.8% to 10.4%, potassium from 261.0 to 355.2 mg/100 g, phosphorus from 242.9 to 338.0 mg/100 g, magnesium from 20.4 to 33.5 mg/100 g, zinc from 1.7 to 3.8 mg/100 g and sodium from 967.0 to 1053 mg/100 g, but reduced Ca. The improved contents fulfilled the requirement of protein and micronutrients in meat emulsion [174]. In conclusion, lean meat can conveniently be replaced with cricket flour. This innovative step may encourage food industries to follow this mixture in food recipes not only to improve current entomophagy practices adopted by tribal communities but also to popularize mixing in commercial products sold by food companies.

## 4. Impact of Insect Quality on Consumers’ Preference and Acceptability

For accepting insects as food, nutrient content (protein being a major component), quality of insects (particularly, taste, flavour, appearance, palatability) and external factors (availability, convenient pricing, conducive social environment) are important [12,175]. Forest-dwelling communities not only in developing countries [17] but also in rural places like, for instance, in Japan [176] have easy access to wild areas and prefer wild insects because of their taste. Currently, little is known about the consumers’ reactions to wild insects and their food products, preference, acceptance and consumption of insect-based foods [29]. There are, however, anecdotal reports of preferences and greater acceptance of selected wild species of edible insects in Africa and India over reared species like silkworms or crickets.

In Kenya, Alemu et al. [177] found no significant difference in consumption of whole or powdered termites. At the local market, consumers checked the insect stock for freshness, presence of legs, cleanliness, species type and oil content before purchasing termites, for example, *Macrotermes falciger* (Ruelle). The majority of the buyers (77.6%) preferred fried adults [178]. Termite soldiers with long bodies were in great demand and highly preferred over alate forms [178]. The grasshopper (*R. differens*) is a traditional delicacy, a source of nutrients and tasty multipurpose insect in Tanzania, Kenya and Uganda [179]. Consumers preferred grasshoppers which were salted, boiled and smoked or deep fried in cotton seed oil over any other single processing methods (smoking, deep frying, sun-drying, toasting, boiling) [179]. In another survey, *R. differens* adults boiled with salt, onion and tomato and then dried, were preferred over those only deep-dried with salt and onion. The acceptability ranking was 7.2 and 5.2 for these products respectively (ranking scale of 0–9, with 9 being the maximum acceptance: [137]). In the case of *R. nitidula,* people in Uganda preferred the boiled and dried grasshoppers with salt, onion and tomatoes over those which were simply boiled and dried without tomatoes. In India whole insects are preferred, with the exception of grasshoppers, whose legs are sometimes removed; larvae, pupae and adult termites are often mixed and sold together by indigenous vendors [13].

Overall, although a greater acceptance was noted for insects without much attention to the species, fear of trying an unknown product, lack of taste experience and a belief of low social acceptance were considered as major constraints in popularizing edible insects [180] even though taste alone in more than 50% of probands tested did not enable them to distinguish insects from cheese or bread [181]. Correct labelling is also an important factor of acceptability. Recently, while assessing the accuracy of insect products in the UK market, Siozios et al. [182] found, by using DNA barcoding, frequent disparities between identity in packages containing mopane caterpillars, winged termites and grasshoppers. This may distract consumers from accepting and consuming insects or insect products.

In selecting an edible species information on entomophagy, prior experience and familiarity with edible insects, appearance, flavour and overall likability of a species are major factors [12]. Therefore, information and knowledge can influence attitudes towards insects as food and food supplemented with edible insects [183]. In fact, Van Thielen et al. [184] reported an increasing positive response in Belgium regarding acceptance, as revealed by a survey undertaken two years after the introduction of edible insects in that country. In a similar survey of Danish consumers revealed the fact that 23% of them were willing to eat insects [185].

Before experiencing the taste of cricket powder, Canadian consumers thought that consumption was undesirable, but their attitude changed after having consumed the powder and they were then willing to buy it [186]. In the USA, Mexico and Spain, replacing wheat flour with 15% and 30% of cricket (*A. domesticus*) powder in chocolate chip cookies was evaluated [187]. No difference between 15% and 30% was noted by USA consumers whilst Mexican and Spanish consumers liked the 15% sample significantly more than the control and 30% sample. From this survey, it was concluded that 15% powder did not negatively impact acceptability but improved liking and protein content in cookies. In Brazil, female consumers were more reluctant towards entomophagy than male subjects, but in Benin no such gender effect existed [188]. Preference was given to whole insects, although insect flour was liked by 40% of all consumers. Generally, insects were considered safe for consumption by educated consumers familiar with entomophagy [189]. Perception of entomophagy by residents of Korea and Ethiopia assessed through structured questionnaires revealed a positive note [190].

According to Medigo et al. [130], Belgians also have a positive attitude towards consuming insects and they have, moreover, developed a taste for certain species. Therefore, Sun-Waterhouse et al. [28] opined that practical approaches for transforming insect biomass into consumer food products are vital. Based on a survey and experiment in Australia and the Netherlands, Lensvelt and Steenbekkers [8] concluded that providing information on entomophagy and giving people the opportunity to try insects were two important aspects to influence consumers’ attitude towards edible insects.

As another approach, Collins et al. [191] suggested educating school children, extending promotion for acceptance of the insect product to facilitate the adoption of insect food as a mainstream item and, thereby, as an available product through market chains. For example, the number of “burgers” with meal worms consumed by western people depended upon appearance, taste and flavour [192]. These criteria are important especially when mealworm products are considered inferior to the carrier products [193]. Medigo et al. [130] found that the processed mealworms with chocolate were the most popular insects whereas whole and crushed mealworms or boiled or baked crickets were least consumed. Similarly, despite the good nutritional qualities of mealworm larvae, it is uncertain that they would become a safe source of protein for Europeans because of their difficult to control, highly and variable microbial load [194].

Edible insects are not only something for developing countries, but equally important for developed countries (because of the problems of the latter with an increasingly obese populace) where they ought to find perhaps even greater acceptance than in the developing countries. Information about presentation, conservation, preservation of food products and local marketing is to some extent now available [17]. Studies are lacking, however, on likely changes in flavour, taste and texture of mealworm and other insect-containing products during storage; for the industrial production, moreover, information on suitable packaging and presentation of insect products is equally important. Effective advertising of edible insects could undoubtedly do with improvements and using catchy slogans like “Forget about the pork and put a cricket on your fork” or “Mealworms and spaghetti is food that makes you happy” could be expected to help as well [30].

## 5. Conclusions and Suggestions for Future Research

Insects are being considered as an alternative to conventional protein sources for both developing as well as developed countries. Edible insects have received attention from researchers in recent years because, firstly, the consumption of insects has spread to urban areas and the current concept is oriented towards health-related as well as ecological issues, and secondly, because conventional livestock rearing and certain systems of crop cultivation have proved environmentally disastrous [27,195,196,197]. Bioprospecting is currently limited to a few eco-zones in countries where insects have commonly been consumed. Intensive surveys may yet reveal more species that could be considered for consumption and farming. The shelf life of processed insects could possibly be improved if more research were devoted to this aspect. Factors responsible for nutrient content and quality of edible insects have not been explored sufficiently and to know how the chemical composition, handling and storage methods, contamination with micro-organisms, the insects’ diet, feeding schedules, host plants and the plant’s own nutrient content as well as the seasons affect food insect marketability would be of considerable benefit in selecting the most suitable species [198].

There is a need to develop rearing facility designs to be made available to small mass production units, which can help create a socially acceptable climate for the expansion of entomophagy [199]. Mass collections of aquatic insects by using nets woven by fishermen may be a remunerative venture, but encouraging locals and creating marketing channels as well as obtaining permits to fish for aquatic insects could be major hurdles. Recently, Oppert et al. [200] suggested sequencinggene transcripts from embryos, one-day hatchlings, nymphs and male and female adults of *A. domesticus* to use genetically modified crickets for improved insect production.

Regulations and legislation along with proper farming procedures, storage and hygiene would benefit consumers by way of healthier insects. Frameworks shared by different countries exist in Europe [201] but are lacking for most developing countries [202]. Proper processing and decontamination methods against micro-organisms during collection and storage, and preference of species should be included in surveys to ensure food safety [22]. A compilation of all information may be used to select a few insect species for mass rearing or augmenting their survival rate in natural habitats and a linkage through regional or international networks among countries/regions where entomophagy is practiced would be a first step. The network could facilitate exchanges and dissemination of information on insects and insect related recipes.

It is essential to conserve wild edible insect populations and to improve the survival of the most popular species [17]. This can be achieved by studying the population dynamics of these insects, identifying their host plants, and controlling their enemies. Other actions can include restrictions on over-harvesting, revoking the decreasing diversity of host plants, boosting the insects’ resilience to adverse weather phenomena and seasonal effects and monitoring insect diseases.

A regulatory legal framework is required to guarantee that manufacturing practices, quality management, hazard analysis and other issues related to content and quality of edible insects are meeting acceptable standards [202]. Furthermore, proper labelling and documentation of the insect product would help to boost the consumers’ knowledge and interest in entomophagy as would some cheeky and witty slogans to promote insect-containing food items [30]. The scientific guidelines explained by the European Food Security Authority [201] are worth studying to prepare a manual for insects consumed in developing countries, either on a regional or national basis to assure food and nutritional security.

In certain regions the people’s diet may lack zinc, but in others there may be a shortage of magnesium, or iron, or calcium. To improve situations such as these, some species of insects could be promoted that are particularly rich in the minerals that are needed. Likewise, there may be reasons to boost certain fatty acids in the diet, acids that could be supplied by specific species of insects. To be able to select the appropriate species, it is of course essential to know precisely the chemical composition of the insect species, which demonstrates how important it is to have a detailed catalogue of the contents of as many species of insects as possible. If one extends this to animal feed, fish culturists may desire in particular protein-rich species, but pigs should perhaps be fed fatty insects and poultry farmers may wish to obtain insects with a high calcium content.

To promote insect-based functional foods as a platform for certain health-related properties is a promising option and is to some extent already taking place, e.g., larvae of the pallid emperor moth *Cirina forda* for protein solubility, oil absorption capacity and foaming stability [203], *T. molitor* larvae for their oil, foaming and emulsion capacity [204], black soldier fly for peptides with antimicrobial activity against the stomach ulcer bacterium *Helicobacter pylori* [205] and male silkworm pupal extract with its Viagra-like effect for erectile dysfunction [206].In fact, Meyer-Rochow [207] reviews hundreds of species that can be used therapeutically, but in many cases also serve as food for humans. This is an aspect certainly worth exploring further.

## Figures and Tables

**Table 1:** Proximate nutrient composition (g/100 g dry matter basis) of edible insects.

Insect	Developmental Stage	Protein	Fat	Fibre	NFE *	Ash	Reference
**Blattodea (including infra order Isoptera)**							
Edible cockroaches and termites		46.3	31.3	5.2	13.7	4.4	[22]
*Macrotermes bellicosus*	A	40.7	44.8	5.3	2.2	5.0	[36]
*Macrotermes nigeriensis*	A	37.5	48.0	5.0	2.1	3.2	[37]
*Odototermes* sp.	A	33.7	50.9	6.3	6.1	3.0	[38]
*Syntermes* sp. soldier	A	64.7	3.1	23.0	2.5	4.2	[36]
**Coleoptera**							
Edible beetles		40.7	33.4	10.7	13.2	5.1	[22]
*Allomyrina dichotoma*	L	54.2	20.2	4.0	17.7	3.9	[39]
*Oryctes rhinoceros*	L	52.0	10.8	17.9	2.0	11.8	[37]
*Protaetia brevitarsis*	L	44.2	15.4	11.1	22.5	6.9	[39]
*Tenebrio molitor*	L	53.2	34.5	6.3	1.9	4.0	
*Tenebrio molitor*	P	51.0	32.0	12.0	--	--	[40]
*Tenebrio molitor*	L	52.0	31.0	13.0	--	--	
*Zophobas morio*	L	46.0	35.0	6.0	--	--	
**Diptera**							
Edible flies		49.5	22.8	13.6	6.0	10.3	[22]
*Caliphora vomitoria*	A	64.9	0.7	16.6	12.2	5.6	[41]
*Hermetia illucens*	Pre P	44.3	31.9	5.1	3.4	8.7	
*Hermetia illuscens*	L	39.0	32.6	12.4	--	14.6	[42]
**Hemiptera**							
Edible bugs		48.3	30.3	12.4	6.1	5.0	[22]
*Aspongopus nepalensis*	A	10.6	38.4	33.5	15.3	2.2	[18]
**Hymenoptera**							
Edible ants, bees, wasps		46.5	25.1	5.7	20.3	3.5	[22]
*Oecophylla smaragdina*	A	55.3	15.0	19.8	7.3	2.6	[38]
**Lepidoptera**							
Edible moth		45.4	27.7	6.6	18.8	4.5	[22]
*Cirina butyrospermi*	L	62.7	14.5	5.0	12.6	5.1	[43]
**Odonata**							
Edible dragonfly, damselfly		55.2	19.8	11.8	4.6	8.5	[22]
**Orthoptera**							
Edible grasshoppers, crickets, locusts		61.3	13.4	9.6	13.0	3.9	[22]
*Acheta domesticus*	A	62.6	12.2	8.0	12.3	5.0	[41]
*Brachytrupes* sp.	A	65.4	11.8	13.3	2.5	4.9	[36]
*Brachytrupes orientalis*	A	65.7	6.3	8.8	15.2	4.3	[44]
*Chondacris rosea*	A	68.9	7.9	12.4	6.7	4.2	
*Gryllus assimilis*	A	56.0	32.0	7.0	--	--	[40]
*Gryllus bimaculatus*	A	58.3	11.9	9.5	10.6	9.7	[39]
*Ruspolia nitidula*	A	40.8	46.3	5.9	3.7	3.3	[41]
*Schistocerca piceifrons piceifrons*	A	80.3	6.2	12.6	--	3.4	[45]
*Teleogryllus emma*	A	55.7	25.1	10.4	0.7	8.2	[39]

L = Larva, P = Pupa, N = Nymph, A = Adult, B = Brood, NFE * = Nitrogen-free extract (indicative of soluble carbohydrates).

**Table 2:** Comparative account of proximate nutrient content (g/100 g dry matter basis) of different species belonging to same genus.

Genus	Species	Developmental Stage	Protein	Fat	Fibre	NFE *	Ash	Reference
**Blattodea**								
*Macrotermes*	*bellicosus*	A	20.4	28.2	2.7	43.3	2.9	[46]
	*notalensis*		22.1	22.5	2.2	42.8	1.9	
	*subhylanus*		39.3	44.8	6.4	1.9	7.6	[47]
	*bellicosus*		39.7	47.0	6.2	2.4	4.7	
*Periplaneta*	*americana*	L,A	65.6	28.2	3.0	0.8	2.5	[48]
	*australasiae*		62.4	27.3	4.5	2.7	3.0	
*Pseudacanthotermes*	*militaris*	A	33.5	46.6	6.6	8.7	4.6	[47]
	*spiniger*		37.5	47.3	7.2	0.7	7.2	
**Coleoptera**								
*Oryctes*	*boas*	L	26.0	1.5	3.4	38.5	1.5	[46]
	*rhinoceros*		42.3	0.6	--	27.7	12.7	[49]
**Hemiptera**								
*Edessa*	*conspersa*	N,A	36.8	45.8	10.0	4.2	3.2	[50] (*cf.* [22])
	*montezumae*		37.5	45.9	10.9	2.1	3.7	
	*petersii*		37.0	42.0	18.0	1.0	2.0	[51]
	sp.		33.0	54.0	11.0	--	1.0	
**Hymenoptera**								
*Atta*	*mexicana*	A	46.0	39.0	11.0	0.0	4.0	[51]
	*cephalotes*		43.0	31.0	10.0	14.0	2.0	
*Brachygastra*	*azteca*	B	63.0	22.0	3.0	9.0	3.0	
	*mellifica*		53.0	30.0	3.0	11.0	3.0	
*Polybia*	*parvulina*	B	61.0	21.0	6.0	8.0	4.0	
	*occidentalis nigratella*		61.0	28.0	2.0	11.0	3.0	
	*occidentalis bohemani*		62.0	19.0	4.0	13.0	3.0	
**Lepidoptera**								
*Anaphe*	*infracta*	L	20.0	15.2	2.4	66.1	1.6	[46]
	*recticulata*		23.0	10.2	3.1	64.6	2.5	
	*venata*		25.7	23.2	2.3	55.6	3.2	
	sp.		18.9	18.6	1.7	46.8	4.1	
**Orthoptera**								
*Sphenarium*	*purpurascens*	A	65.2	10.8	9.4	11.6	3.0	[48]
	*mexicanum*		62.1	10.8	4.1	22.6	0.3	
	*purpurascens*		56.0	11.0	9.0	21.0	3.0	[51]
	*histrio*		77.0	4.0	12.0	4.0	2.0	
	sp.		68.0	12.0	11.0	5.0	5.0	

L = Larva, P = Pupa, N = Nymph, A = Adult, B = Brood, NFE * = Nitrogen-free extract (indicative of soluble carbohydrates).

**Table 3:** Amino acid composition of different species belonging to the same genus.

Genus	Species	Amino Acid Composition (% of Total Amino Acids or Protein)																		Total Amino Acids or Protein (g/100 g Dry Matter)	Reference
		Val	Ile	Leu	Lys	Tyr	Thr	Phe	Trp	His	Met+Cys	Total EAA ^††^	Arg	Asp	Ser	Glu	Gly	Ala	Pro		
*Apis* * (P)	*mellifera*	5.9	5.6	7.8	7.3	4.9	4.6	0.5	ND	2.7	1.0	40.3	5.6	8.6	4.9	20.5	6.1	7.1	ND	40.9	[33]
	*cerana*	6.1	4.7	8.6	5.9	3.7	4.3	4.1	ND	2.5	4.7	44.6	4.9	12.3	4.7	10.4	7.2	9.6	6.6	51.2	[34]
	*dorsata*	5.7	4.4	8.5	5.7	3.3	4.4	3.9	ND	2.6	4.9	43.4	4.9	13.4	4.9	11.1	7.5	8.5	6.9	38.9	
	*florea*	5.9	4.8	9.3	6.5	4.5	4.8	4.8	ND	2.8	4.8	48.2	5.3	10.4	5.1	14.0	6.2	8.1	7.6	35.6	[35]
*Bombus* * (A)	*ignitus*	7.0	5.7	9.3	6.1	3.0	2.3	2.7	ND	3.0	6.1	45.2	4.0	3.8	4.9	11.4	9.1	11.2	10.1	47.3	[52]
	*terrestris*	6.3	5.0	8.1	7.8	3.1	2.3	3.1	ND	2.6	6.3	44.6	5.0	3.9	6.3	12.5	8.1	10.2	9.9	38.3	
*Brachygastra* (B)	*azteca*	6.4	5.1	8.5	6.1	6.5	4.4	4.1	0.7	2.8	3.0	47.6	4.4	8.4	4.5	16.4	6.7	5.8	6.4	63.0	[51]
	*mellifica*	5.4	4.4	7.8	3.6	7.5	4.4	4.0	0.7	3.6	3.8	45.2	5.7	8.6	4.2	16.0	6.7	6.1	7.1	53.0	
*Polybia* (B)	*occidentalis nigratella*	5.9	4.5	7.8	7.4	5.6	4.0	3.3	0.7	3.0	5.0	47.2	5.7	8.4	4.5	12.9	7.1	6.5	6.3	61.0	
	*parvulina*	6.1	4.7	7.8	7.3	5.9	4.1	3.4	0.7	3.4	5.3	48.7	5.7	7.8	4.4	13.3	7.2	6.4	6.5	61.0	
*Polistes* *	*sagittarius*	6.6	5.5	7.8	4.4	5.0	4.2	5.0	ND	3.0	1.4	42.9	4.4	8.3	4.4	17.2	6.9	7.2	8.9	36.1	[31]
	*sulcatus*	6.7	6.2	8.0	4.2	4.9	4.2	4.4	ND	2.4	2.0	43.0	4.0	7.3	4.4	15.3	8.9	8.9	8.0	45.0	
*Vespa* * (B)	*velutina*	6.1	5.5	8.7	6.1	6.6	4.2	4.2	ND	3.2	2.4	47.0	4.5	6.3	4.5	20.1	6.3	5.5	6.1	37.9	[32]
	*mandarinia*	6.3	5.7	8.7	6.3	7.3	4.3	4.3	ND	3.3	2.7	48.9	2.2	6.5	4.3	21.2	6.3	5.4	5.7	36.8	
	*basalis*	5.7	5.3	8.5	6.8	7.1	4.3	4.3	ND	3.2	1.4	46.6	4.3	6.4	4.3	22.1	5.7	5.0	5.7	28.1	
*Vespa* * (L)	*basalis*	5.9	5.9	8.0	4.3	5.7	4.1	4.3	ND	2.5	2.1	42.8	3.9	7.7	4.3	17.1	8.2	7.7	8.4	43.9	[31]
	*mandarinia mandarinia*	5.0	4.6	6.1	16.5	4.0	3.3	10.5	ND	2.1	0.8	52.9	3.3	6.3	3.4	13.2	6.3	6.5	7.9	52.2	
	*velutina auraria*	6.9	5.9	7.6	2.9	7.6	4.3	4.1	ND	3.1	2.9	45.3	6.3	9.2	6.5	12.0	8.0	7.1	5.9	49.0	
	*tropica duealis*	7.5	5.4	8.3	3.3	5.4	4.5	4.2	ND	1.4	1.2	41.2	7.1	10.1	5.0	13.4	8.7	7.8	6.6	42.4	
*Sphenarium*	*histrio*	5.1	5.3	8.7	5.7	7.3	4.0	11.7	0.6	1.9	3.3	53.6	6.6	9.3	5.1	5.3	5.3	7.6	7.2	77.0	[51]
	*purpurascens*	5.7	4.2	8.9	5.7	6.3	3.1	10.3	0.7	2.2	4.3	51.4	6.0	8.7	4.8	10.7	6.8	6.4	6.2	56.0	

L = Larva, P = Pupa, A = Adult, B = Brood; ND = Not determined or not estimated; * Amino acid content (g/100 g dry matter) was obtained from the respective paper and recalculated as g/100 g of total amino acids or protein; ^††^ EAA: Essential amino acids, we include essential amino acids (Val, Ile, Leu, Lys, Thr, Trp, Phe, His, Met) and two conditional essential amino acids (Tyr, Cys).

**Table 4:** Fatty acid composition of selected edible insects.

Genus	Species	Developmental Stage	Fatty Acid Composition (% of Total Fatty Acids)								Total Fatty Acids or Fat (g/100 g Dry Matter)	Reference
			C14:0	C16:0	C18:0	SFA	C18:1	MUFA	C18:2	PUFA		
*Apis* ^†^	*cerana*	L	3.9	38.2	8.1	50.7	46.9	48.7	0.5	0.7	6.1	[34]
		P	3.0	31.4	10.6	46.2	49.8	52.7	0.9	1.1	6.3	
		A	1.9	18.2	12.1	33.8	57.7	63.4	2.6	2.8	4.2	
	*dorsata*	P	3.2	33.3	11.8	49.4	47.7	49.8	0.8	0.8	6.2	
		A	1.0	14.4	14.4	31.3	61.0	66.5	2.2	2.2	3.1	
	*mellifera*	L	2.4	37.3	11.8	51.8	47.5	48.2	0.0	0.0	4.9	[33]
		P	2.9	35.1	12.6	51.1	47.6	48.9	0.0	0.0	5.5	
		A	0.6	14.4	9.3	25.2	45.2	67.0	7.8	7.8	1.7	
	*florea*	P	1.8	35.3	8.8	46.6	47.6	52.3	1.0	1.1	7.2	[35]
		A	1.5	30.7	9.7	43.2	49.7	55.7	1.1	1.1	5.4	
*Aspongopus*	*viduatus*	A	0.3	31.3	3.5	37.9	45.5	56.8	4.9	5.4	54.2	[53]
	*nepalensis*	A	0.4	32.3	4.8	37.5	46.4	56.1	6.1	6.1	35.9	[18]
*Bombus* *^,†^	*ignitus*	A	2.6	16.1	1.7	22.1	49.1	75.4	2.5	2.5	9.5	[52]
	*terrestris*	A	3.8	15.2	1.7	21.5	51.1	76.2	2.2	2.2	8.4	
*Imbrasia*	*belina*	L	1.2	31.9	4.7	37.9	34.2	36.0	6.0	26.1	23.4	[54]
	*epimethea*	L	0.6	23.2	22.1	46.1	8.4	9.0	7.0	42.5	13.3	[22]
	*truncata*	L	0.2	24.6	21.7	46.5	7.6	7.6	7.6	44.4	16.4	
	*ertli*	L	1.0	22.0	0.4	61.4	2.0	24.0	20.0	31.0	11.1	[55,56]
	*oyemensis*	L	0.5	46.0	7.2	54.2	34.6	34.6	11.2	11.2	25.4	[22]
*Macrotermes*	*Bellicosus* **	A	2.2	42.5	2.9	49.0	15.8	17.9	24.2	33.1	36.1	[54]
	*bellicosus*	A	0.2	46.5	--	46.7	12.8	14.9	34.4	38.3	46.1	[56,57]
	*nigeriensis*	A	0.6	31.4	7.1	39.4	52.5	53.1	7.6	7.6	34.2	[58]
	*subhylanus*	A	1.1	27.7	6.3	35.1	48.6	52.8	10.8	12.2	44.8	[47]
	*bellicosus*	A	1.2	38.4	9.5	49.5	41.7	44.6	5.0	5.9	47.0	
*Pseudacanthotermes*	*militaris*	A		26.0	5.9	32.2	50.3	56.1	11.5	11.7	46.6	
	*spiniger*	A	0.8	28.0	6.1	35.8	49.3	52.9	10.5	11.3	47.3	
*Oryctes*	*owariensis*	L	2.5	0.2	0.2	3.1	5.2	43.6	45.5	50.9	53.8	[59]
	*rhinoceros*	L	3.5	28.7	2.1	34.4	41.5	45.9	14.1	19.7	38.1	[54]
*Vespa* ^†^	*velutina*	B	6.0	31.9	7.8	48.3	35.3	39.7	5.2	12.1	11.6	[32]
	*mandarinia*	B	2.5	21.3	5.0	30.7	27.7	29.2	33.7	40.1	20.2	
	*basalis*	B	1.4	15.8	5.4	24.3	23.9	25.2	42.8	50.5	22.2	

L = Larva, P = Pupa, A = Adult; ^†^ Fatty acid content (mg/100 g dry matter) was obtained from the respective paper and recalculated as % of total fatty acids; * Mated queen; ** Oil. SFA = Saturated fatty acids, MUFA = Monounsaturated fatty acids, PUFA = Polyunsaturated fatty acids

**Table 5:** Minerals content (mg/100 g) of selected edible insects.

Genus	Species	Developmental Stage	Ca	Mg	Na	K	P	Fe	Zn	Cu	Mn	Reference
*Anaphe*	*infracta*	L	8.6	1.0			111.3	1.8				[46]
	*reticulate*	L	10.5	2.6			102.4	2.2				
	*venata*	L	8.6	1.6			100.5	2.0				
	sp.	L	7.6	1.0			122.2	1.6				
	*venata*	L	40.0	50.0	30.0	1150.0	730.0	10.0	10.0	1.0	40.0	[60]
*Apis*	*cerana*	L	63.1	86.6	37.2	823.1	715.6	5.9	7.3	1.0	1.1	[34]
		P	62.9	104.3	44.4	1153.2	931.5	7.1	7.7	1.2	0.2	
		A	91.1	148.8	77.1	1538.8	1283.9	11.1	12.9	1.9	0.2	
	*dorsata*	P	68.9	103.4	48.6	1136.6	905.0	5.8	6.4	1.1	0.1	
		A	78.5	113.3	53.9	1254.3	972.3	7.6	7.4	1.2	0.1	
*Brachytupes*	*orientalis*	A	76.3	87.2	112.0	412.3		18.7	8.5	1.5	5.0	[44]
	sp.	A	9.2	0.1			126.9	0.7				[22]
*Imbrasia*	*epimethea*	L	224.7	402.2	75.3	1258.1	666.7	13.0	11.1	1.2	5.8	[22]
	*ertli*	L	55.0	254.0	2418.0	1204.0	600.0	2.1		1.5	3.4	
	*oyemensis*	L	73.0		730.0	680.0						
*Macrotermes*	*subhylanus*	A	58.7					53.3	8.1			[47]
	*bellicosus*	A	63.6					116.0	10.8			
*Pseudacanthotermes*	*militaris*	A	48.3					60.3	12.9			
	*spiniger*	A	42.9					64.8	7.1			

L = Larva, P = Pupa, A = Adult.

**Table 6:** Comparative account of proximate nutrient content (g/100 g dry matter basis) of different developmental stages of edible insects.

Insect	Developmental Stage	Protein	Fat	Fibre	NFE *	Ash	Reference
**Coleoptera**							
*Tebebrio molitor*	L	47.7	37.7	5.0	7.1	3.0	[66]
	P	53.1	36.7	5.1	1.9	3.2	
	A	60.2	20.8	16.3	0.01	2.7	
*Rhynchophorus phoenicis*	Early L	9.1	61.5	22.1	4.9	2.4	[67]
	Late L	10.5	62.1	17.2	7.8	2.3	
	A	8.4	52.4	21.8	16.0	1.4	
*Rhynchophorus phoenicis*	L	23.4	54.2	3.4	5.0	5.2	[68]
	Immature P	33.1	42.7	3.1	6.7	7.4	
	Mature P	34.9	47.1	2.4	5.6	3	
	A	34.1	44.7	7.2	4.0	5.8	
*Rhynchophorus phoenicis*	Early L	9.1	24.2	5.8	13.0	2.4	[69]
	Late L	10.5	25.4	6.0	12.0	2.3	
*Oryctes rhinoceros*	L	70.8	7.5	5.4	7.0	8.3	[70]
	P	65.3	20.2	2.2	4.3	3.2	
	A	74.2	9.6	3.7	2.8	5.3	
**Hymenoptera**							
*Apis mellifera*	L	42.0	19.0	1.0	35.0	3.0	[51]
	P	49.0	20.0	3.0	24.0	4.0	
*Apis mellifera ligustica*	L	35.3	14.5		45.1	4.1	[33]
	P	45.9	16.0		34.3	3.8	
	A	51.0	6.9		30.5	11.5	
**Orthoptera**							
*Acheta domesticus* (as is basis)	N	15.4	3.3	5.8	0.9	1.1	[71]
	A	20.5	6.8		10.0	1.1	
*Zonoceros variegatus*	N1	18.3	4.3	0.9	0.4	1.9	[62]
	N2	14.4	4.8	0.9	0.4	1.0	
	N3	16.8	2.9	1.5	0.9	0.9	
	N4	15.5	0.7	0.9	9.7	1.6	
	N5	14.6	1.1	0.9	9.8	1.6	
	N6	16.1	0.9	1.0	8.8	1.5	
	A	21.4	0.9	1.2	10.0	1.4	

L = Larva, P = Pupa, N = Nymph, A = Adult; NFE * (nitrogen-free extract) indicates carbohydrate.

**Table 7:** Anti-nutrient content (mg/100 g) of selected edible insects.

	Phytate	Tannin	Oxalate	Trypsin Inhibitor	Lectin	Hydrocyanide	Reference
Ant ^†^	2030.8	400.0					[143]
Termite ^†^	2482.1	948.3					
Winged termite ^†^	1128.2	250.0					
Cricket ^†^	3159.0	900.0					
Meal bug	2256.4	1150.0					
Grasshopper ^†^	1100.1	1050.0					
*Anaphe venata* ^†^	1918.0	753.3					
Tree hopper		1000.0					
*Rhynchophorus pheonicis* * L	1.4	1.0	0.1	0.9	0.6		[144]
*Gymnogryllus lucens*^†^ A	0.03	0.03	1.3			0.2	[145]
*Heteroligus meles* ^†^	0.03	0.04	2.8			0.3	
*Rhynchophorus*^†^ L	0.03	0.04	1.8			0.2	
*Zonocerus variegatus*^†^ A	0.03	0.04	2.6			0.3	
*Oedaleus abruptus*^†^ A		2450.0	600.0				[146]
*Lethocerus indicus* * N,A		372.3					[147]
*Laccotrephes maculatus* * N,A		350.4					
*Hydrophilus olivaceous* * A		528.7					
*Cybister tripunctatus* * A		301.7					
*Crocothemes servillia* * N		465.3					
*Macrotermes nigeriensis*^†^ A	15.2	0.6	103.0				[37]
*Oryctes rhinoceros*^†^ L	16.1	0.6	109.0				
*Oecophylla smaragdina*^†^ A	171.0	496.7					[38]
*Odontotermes* sp. ^†^ A	141.2	615.0					
*Oxya hyla hyla*^†^ A		2316.0	474.0				[148]
*Oryctes rhinoceros*^†^ L	37.0	5.6	1.3				[70]
*Oryctes rhinoceros*^†^ P	39.4	6.8	1.3				
*Oryctes rhinoceros*^†^ A	41.1	4.2	1.2				

L = Larva, P = Pupa, N = Nymph, A = Adult; * Anti-nutrient content was estimated on the basis of wet weight; ^†^ Anti-nutrient content was estimated on the basis of dry weight.

## Data Availability

Not applicable.
